# Historical Overview of the Evolution of Multidrug-Resistant Gram-Negative Infections in Tunisia from 1999 to 2019

**DOI:** 10.3390/antibiotics14070657

**Published:** 2025-06-29

**Authors:** Lamia Kanzari, Sana Ferjani, Basma Mnif, Faouzia Mahjoubi, Mariem Zribi, Khaoula Meftah, Asma Ferjani, Emna Mhiri, Yomna Ben Lamine, Yosr Kadri, Habiba Naija, Manel Hamdoun, Yosra Chebbi, Sarra Dhraief, Naglaa Mohamed, Hela Zaghden, Lamia Thabet, Wafa Achour, Olfa Bahri, Farouk Barguellil, Maha Mastouri, Sophia Besbes, Leila Slim, Hanen Smaoui, Adnene Hammami, Ilhem Boutiba-Ben Boubaker

**Affiliations:** 1 National Reference Laboratory on Antimicrobial Resistance Surveillance, Tunis 1006, Tunisia; ferjsana@yahoo.fr (S.F.); aferjani76@gmail.com (A.F.); ilhem.boutiba@gmail.com (I.B.-B.B.); 2 Microbiology laboratory, Charles Nicolle Hospital, Tunis 1006, Tunisia; 3 Research Laboratory Antimicrobial Resistance LR99ES09, Faculty of Medicine of Tunis, University of Tunis El Manar, Tunis 1007, Tunisia; 4 Laboratory of Microbiology of Sfax University Hospital Center, Sfax 3000, Tunisia; basmamnif@gmail.com (B.M.); mahjoubi_faouzia@medecinesfax.org (F.M.); hammami.adnene@gmail.com (A.H.); 5 Research Laboratory LR03SP03 “Micro-Organisme et Pathologie Humaine”, Faculty of Medicine, University of Sfax 3000, Tunisia; 6 Laboratory of Microbiology, La Rabta Hospital, Tunis 1007, Tunisia; meriamzribimiled@gmail.com; 7 Laboratory of Microbiology, Bechir Hamza Children’s Hospital, Tunis 1006, Tunisia; meftahkhaoula@gmail.com (K.M.); hanen.smaoui@gmail.com (H.S.); 8 Laboratory of Microbiology, Abderrahmen Mami Hospital, Ariana 2080, Tunisia; emna.mehiri@tunet.tn (E.M.); leilaslimsaidi@gmail.com (L.S.); 9 Laboratory of Microbiology, Mohamed Kassab Orthopaedics Institute, La Manouba 2010, Tunisia; yomnabenlamine@gmail.com (Y.B.L.); sophia.besbes@yahoo.fr (S.B.); 10 Laboratory of Microbiology, Fatouma Bourguiba Hospital, Monastir 5000, Tunisia; yosrguedri@gmail.com (Y.K.); mastourimaha@yahoo.fr (M.M.); 11 Laboratory of Transmissible Diseases and Biologically Active Substances LR99ES27, Faculty of Pharmacy, University of Monastir, Monastir 5000, Tunisia; 12 Laboratory of Microbiology, Military Hospital, Tunis 1008, Tunisia; naijahabiba@gmail.com (H.N.); farouk.barguellil@gmail.com (F.B.); 13 Laboratory of Clinical Biology, Aziza Othmana Hospital, Tunis 1008, Tunisia; hamdoun.zahmoul@gmail.com (M.H.); olfa.bahri@fmt.utm.tn (O.B.); 14 Laboratory Department, Bone and Marrow Transplantation Center, Tunis 1006, Tunisia; yos.chebbi@gmail.com (Y.C.); wafaachour@gmail.com (W.A.); 15 Laboratory of Clinical Biology, Burn and Trauma Center, Ben Arous 2013, Tunisia; dhraiefsarra@gmail.com (S.D.); thabetlamia@gmail.com (L.T.); 16 Pfizer Inc., New York, NY 10001-2192, USA; naglaa.mohamed@pfizer.com; 17 Pfizer Inc., Tunis 1053, Tunisia; hela.zaghden@pfizer.com; 18 Faculty of Pharmacy, University of Monastir, Monastir 5000, Tunisia

**Keywords:** Gram-negative bacteria, antibiotics, antimicrobial resistance, disk diffusion method, Tunisian hospitals, evolution, Tunisia

## Abstract

**Background/Objectives**: As antimicrobial resistance patterns of Gram-negative bacteria change over time, this study aimed to analyze the antimicrobial susceptibility trends of *Escherichia coli*, *Klebsiella pneumoniae*, *Pseudomonas aeruginosa*, and *Acinetobacter baumannii* isolates in Tunisia. **Methods**: From 1999 to 2019, non-duplicate isolates of Gram-negative bacteria were collected from 11 Tunisian hospitals as part of an antimicrobial resistance surveillance program. Antimicrobial susceptibility testing was performed using the disk diffusion method according to the European Committee on Antimicrobial Susceptibility Testing guidelines. **Results**: Out of 213,434 isolates collected during the study period, 58.8% were *E. coli*, 22% were *K. pneumoniae*, 14.4% were *A. baumannii*, and 4.8% were *P. aeruginosa*, with 67% of the isolates sourced from urine samples. *E. coli* showed a significant increase in resistance to third-generation cephalosporins (3GC), from 5.4% in 2004 to 16.5% in 2019, but *K. pneumoniae* displayed a rising trend of resistance to imipenem, from 1% in 2005 to 18.6% in 2019; meanwhile, amikacin remained effective against *K. pneumoniae* isolates. *P. aeruginosa* did not exhibit a significant change in resistance to imipenem. *A. baumannii* had a high resistance rate to imipenem that increased from 34.5% in 2008 to 84.2% in 2019 and had low susceptibility rates to all other antibiotics tested. **Conclusions**: This study reveals high carbapenem resistance among *K. pneumoniae* and *A. baumannii* in Tunisia. *A. baumannii* shows alarming multidrug resistance that requires urgent control measures.

## 1. Introduction

Antimicrobial resistance (AMR) is a serious global public health concern and one of the biggest challenges humans face today. Multidrug-resistant (MDR) bacteria are spreading across the globe at a rate far faster than new antibiotics are being developed [1]. The World Health Organization (WHO) published a list of antimicrobial-resistant pathogens and classified them based on a priority for which new agents are urgently required. Gram-negative bacteria (GNBs) are of significant interest, as three GNBs (namely carbapenem-resistant *Enterobacterales*, carbapenem-resistant *A. baumannii*, and carbapenem-resistant *P. aeruginosa*) are of the highest priority and classified as critical [2]. The 2021–2022 antibiotic resistance-threats report by the Centers for Disease Control and Prevention (CDC) included seven pathogens that require immediate attention, of which four are GNBs [3]. *Enterobacterales* producing extended-spectrum β-lactamases (ESBLs), enzymes that hydrolyze extended-spectrum cephalosporins, are now more prevalent. Carbapenems are the alternative treatment option for ESBL-producing *Enterobacterales*. However, over the past few years, carbapenem resistance is on the rise, and new classes of antimicrobial agents are needed [4]. Misuse of antibiotics, in addition to the situation of MDR, has limited the therapeutic options for many infections to an alarming level [5].

MDR-GNB has been implicated in healthcare-associated infections, as well as community-acquired infections [1]. The most common infections caused by GNBs are bloodstream infections (BSIs), urinary tract infections (UTIs), respiratory tract infections (RTIs), surgical-site infections, and intra-abdominal infections (IAI) [6]. *E. coli*, *K. pneumoniae (Enterobacterales)*, *A. baumannii*, and *P. aeruginosa* (non-fermenting GNBs) are frequently involved in these infections [7].

GNBs pose significant clinical challenges, primarily due to their inherent resistance to numerous antimicrobials, a consequence of their complex cell wall structure, and their exceptional capacity to acquire and disseminate additional resistance mechanisms [8]. This results in a substantial prevalence of MDR, extensively drug-resistant, and occasionally pandrug-resistant infections, which severely restrict therapeutic options and contribute to increased patient morbidity, mortality, and healthcare costs [9]. Clinically, these infections frequently present as severe and difficult-to-treat conditions, such as hospital-acquired pneumonia, bloodstream infections, complicated urinary tract infections, and intra-abdominal infections, particularly in immunocompromised or critically ill patients [10]. Current treatment strategies increasingly depend on last-resort antibiotics, including carbapenems, newer beta-lactam/beta-lactamase inhibitor combinations (e.g., ceftazidime–avibactam, meropenem–vaborbactam, and imipenem–cilastatin–relebactam), cefiderocol, and older agents like colistin and fosfomycin, often employed in combination. This crisis prompts the development of novel therapeutics, including antibiotics, phage therapy, monoclonal antibodies, and innovative approaches to overcome resistance, offering potential solutions [11].

In the Middle East and North Africa region, Tunisia stands out for its high antibiotic consumption, with 38 defined doses administered per 1000 individuals daily. This consumption rate is only exceeded by Greece in the region and is about four times the global average [12]. In 2022, antibiotic use in a major Tunisian teaching hospital was nearly evenly divided between the “Access” (49.33%) and “Watch” (46.89%) groups, with a notable increase in the “Reserve” group (3.77%), indicating rising use of last-resort antibiotics [13]. The extensive use of antibiotics in Tunisia has contributed to the emergence and spread of antibiotic-resistant bacteria such as ESBL-producing *Enterobacterales*, a trend observed in increasing incidence rates in both hospital and community settings, and correlated with national antibiotic consumption data [13,14,15].

The prevalence of ESBL-producing *Enterobacterales* has risen sharply in Tunisia over the past three decades, affecting humans, animals, food, and the environment [16,17]. In urinary tract infections, 57% of third-generation cephalosporin-resistant *Enterobacterales* were ESBL producers, mainly *E. coli* and *K. pneumoniae*, with high resistance to commonly used antibiotics [16]. ESBL-producing *Enterobacterales* have been detected in various food and agricultural environments across Tunisia. Research indicates that these bacteria are present in vegetables (8.2%), soil (7.3%), and irrigation water sourced directly from farms (15.8%), as well as in vegetables available for sale in markets (8.9%), chicken (22.4% of feces and 63.8% of meat samples), and seafood (1.4% in fish and 1.6% in clams) [17,18,19]. The presence of MDR strains and mobile genetic elements in these bacteria raises concerns about the spread of antimicrobial resistance from food sources to the community [20,21].

Since 2006, several carbapenemase-producing GNBs, as well as carbapenem variants, have been detected in Tunisia [22]. Due to the lack of new agents to combat AMR, it is important to monitor the regional prevalence and antimicrobial resistance patterns, which can form the basis for effective empiric treatment. Surveillance programs help guide empiric therapy by providing useful data concerning organism distribution and resistance patterns [23].

In Tunisia, an antimicrobial resistance surveillance program called “L’AntibioRésistance en Tunisie” (LART) was designed in 1999 to track the evolution of antimicrobial resistance and detect the emergence of new resistance phenotypes [24]. In this study, we analyzed the distribution, prevalence, and susceptibility profiles of *E. coli*, *K. pneumoniae*, *P. aeruginosa*, and *A. baumannii* isolates in Tunisia. The data on isolates were recovered from the LART surveillance period between 1999 and 2019. Antimicrobial susceptibility trends were also evaluated using samples collected from hospitalized and outpatients in Tunisia included in the LART database.

## 2. Results

### 2.1. Distribution of Gram-Negative Bacilli

Data on a total of 213,434 *E. coli*, *K. pneumoniae*, *A. baumannii*, and *P. aeruginosa* isolates were collected between 1999 and 2019, of which 58.8% were *E. coli*, 22% were *K. pneumoniae*, 14.4% were *P. aeruginosa*, and 4.8% were *A. baumannii*. The isolates were mainly collected from urines (67%), pus (11.5%), pulmonary samples (7.9%), blood cultures (6.6%), and punctures (1.8%) (Table 1).

### 2.2. Overall Frequency of Antibiotic Resistance

The overall frequency of resistance of *E. coli* and *K. pneumoniae* isolates to a wide range of antibiotics is summarized in Table 2. Overall, among the tested antimicrobial agents, *E. coli* and *K. pneumoniae* were most susceptible to imipenem (IMP) (carbapenems), followed by ertapenem (ETP), piperacillin–tazobactam (TZP), amikacin (AMK) (aminoglycoside), and cefoxitin (FOX) (cephalosporin). *E. coli* isolates showed higher susceptibility to cefotaxime (CTX), ceftazidime (CAZ), and gentamicin (GEN) compared to *K. pneumoniae* isolates.

For non-fermenting GNBs, *P. aeruginosa* isolates had a more than 50% susceptibility rate against the tested antibiotics. They were most susceptible to CAZ and AMK, whereas *A. baumannii* isolates were resistant to most of the tested antimicrobial agents (Table 3).

### 2.3. Antibiotic Resistance Frequency by Samples and Type of Resistant Strains

Antibiotic resistance frequencies of *E. coli* and *K. pneumoniae* were analyzed in urine samples and blood cultures (Table 2). The susceptibility trend for *E. coli* and *K. pneumoniae* isolates in urine samples was similar to overall antibiotic susceptibility. *E. coli*, when isolated from blood cultures, showed a lower susceptibly for CTX and CAZ compared to overall susceptibility. Lower susceptibility rates were observed for all tested antibiotics, except for IMP, for *K. pneumoniae* isolates collected from blood cultures compared to isolates collected from urine samples and overall susceptibility.

Table 3 describes the antibiotic resistance frequencies of *P. aeruginosa* and *A. baumannii* in pulmonary samples and blood cultures. The antibiotic susceptibility profiles of *P. aeruginosa* and *A. baumannii* were similar for pulmonary samples and blood cultures.

### 2.4. Antimicrobial Susceptibility Trends

**Figure 1:**
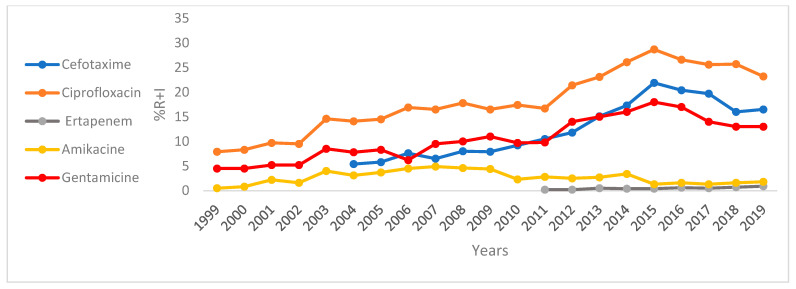
Trend of antibiotic resistance (R + I) of *E. coli* in Tunisia from 1999 to 2019. Resistant (R), Intermediate (I).

**Figure 2:**
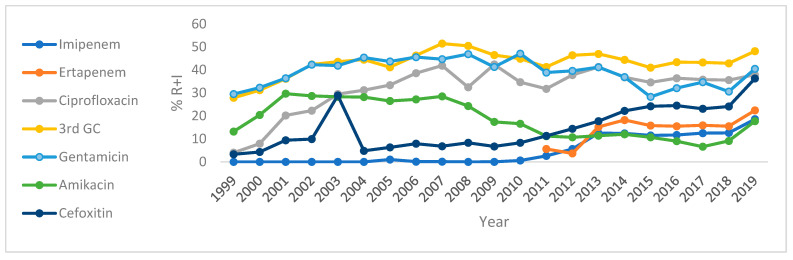
Trend of antibiotic resistance (R + I) of *K. pneumoniae* in Tunisia from 1999 to 2019. Resistant (R). Intermediate (I).

Figure 1 and Figure 2 and Supplementary Tables S1 and S2 depict the trends of resistance plus intermediate resistance of *E. coli* and *K. pneumoniae* to a selected number of antibiotics over the period 1999 to 2019. *E. coli* isolates were highly susceptible to IMP, AMK, and FOX throughout the study period (Table S1). However, susceptibility towards ciprofloxacin, cefotaxime, and gentamicin has been decreasing over the last few years (Figure 1). A statistically significant (*p* < 0.05) correlation was observed between *E. coli* and its resistance to CTX (r = 0.91, from 5.4% in 2004 to 16.5% in 2019), CIP (r = 0.93, from 7.9% in 1999 to 23.2% in 2019), and ERT (r = 0.89, from 0.2% in 2012 to 0.9% in 2019) during this period.

Over the last years, susceptibility of *K. pneumoniae* isolates to IMP, FOX, ciprofloxacin (CIP), and nalidixic acid (NA) has shown a decreasing trend (Table S2).

Figure 2 and Table S2 depict the trends of resistance plus intermediate resistance of *K. pneumoniae* to a selected number of antibiotics, namely CTX, IMP, ERT, CIP, GEN, and AMK, over the period from 1999 to 2019. Resistance of *K. pneumoniae* showed a very strong positive correlation (*p* < 0.005) to IMP (r = 0.92, from 1% in 2005 to 18.6% in 2019) and ERT (r = 0.78, from 5.6% in 2011 to 22.4% in 2019); and a strong positive correlation to FOX (r = 0.72, from 27.9% in 1999 to 48.2% in 2019) and CIP (r = 0.63, from 4% in 1999 to 37.9% in 2019). However, the resistance of *K. pneumoniae* to AMK showed a strong negative correlation, which was statistically significant (r = −0.74, *p* < 0.05).

Overall third-generation cephalosporin (3GC)-resistant *E. coli* strains had lower susceptibility towards all tested antibiotics, except FOX, ETP, IMP, and AMK. For 3GC-resistant *K. pneumoniae*, the resistance rate against FOX, ETP, and IMP has been increasing since 2013. However, the susceptibility of 3GC- and ERT-resistant *K. pneumoniae* strains against AMK has been variable throughout the study period. It varied from 26.2% in 2011 to 33.7% in 2019 for 3GC-resistant *K. pneumoniae*, and from 30.2% in 2012 to 61.5% in 2019 for ERT-resistant *K. pneumoniae*. Overall ERT-resistant strains had lower susceptibility towards all tested antibiotics (Table S5). Colistin (COL) and tigecycline (TIG) maintained their effectiveness against ERT-resistant *K. pneumoniae* and *E. coli* strains. However, their resistance rates for the period 2017–2019 seem to be increasing. TIG resistance rates varied from 2.7% to 20.9% for ERT-resistant *K. pneumoniae* strains and from 0% to 3.9% for ERT-resistant *E. coli* strains. For COL, it varied from 1.7% to 9.9% for ERT-resistant *K. pneumoniae* strains, and from 0% to 11.8% for ERT-resistant *E. coli* strains.

**Figure 3:**
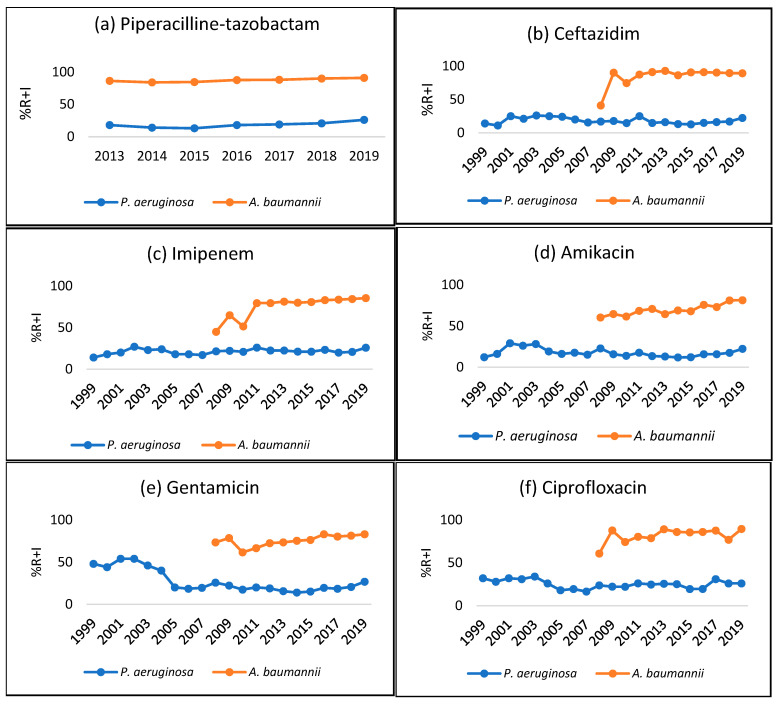
Trend of antibiotic resistance (R + I) of *P. aeruginosa* (from 1999–2019) and *A. baumannii* (from 2008 to 2019) in Tunisia.

Figure 3 depicts the trends of resistance plus intermediate resistance of *P. aeruginosa* (1999–2019) and *A. baumannii* (2008–2019) to TZP, CAZ, IMP, GEN, AMK, and CIP. The increase in *P. aeruginosa* resistance to TZP from 18% in 2013 to 26% in 2019 was statistically significant (r = 0.81, *p* < 0.05); however, resistance to GEN decreased significantly from 48% in 1999 to 26.8% in 2019 (r= −0.6, *p* < 0.05). Correlation between *P. aeruginosa* and its resistance to CAZ (r = −02), IMP (r = 0.28), AMK (r = 0.86), and CIP (r = 0.42) over the period 1999–2019 was not found to be statistically significant (*p* > 0.05).

Concerning *A. baumannii*, a positive correlation was observed between *A. baumannii* and its resistance to IMP (r = 0.95), GEN (r = 0.74), and AMK (r = 0.86) over the period 2008–2019 and TZP (r = 0.89) over the period 2013–2019, which was statistically significant (*p* < 0.05).

In general, IMP-resistant *P. aeruginosa* and *A. baumannii* showed high resistance rates against all tested antibiotics; however, the resistance rate of *A. baumannii* was significantly higher compared to that of *P. aeruginosa* (Table S6). Those strains remain susceptible to COL (3.4% of IMP-resistant *A. baumannii* and 11.5% of IMP-resistant *P. aeruginosa*).

## 3. Discussion

In recent decades, the incidence of MDR organisms has been rising considerably in Tunisia, particularly in GNBs, which are frequently observed in clinical settings. As the antimicrobial susceptibility patterns of GNBs change over time, it is crucial to have real-time country-specific surveillance data to understand the impact of these infections and to guide preventive efforts [25]. This report evaluated the antimicrobial susceptibility trends of *E. coli*, *K. pneumoniae*, *P. aeruginosa*, and *A. baumannii* isolates in Tunisia between 1999 and 2019, using data from LART surveillance program.

*E. coli* were the most commonly encountered GNBs (58.8%) which were predominantly implicated in UTIs, followed by BSIs. The findings are consistent with Guermazi-Toumi et al.’s [26] study conducted in Tunisia. During the study period, *E. coli* strains had high resistance rates for amoxicillin (AMX) (60%), amoxicillin–clavulanic acid (AMC) (25%), and trimethoprim sulfamethoxazole (SXT) (34%). The increasing resistance of *E. coli* to CIP has emerged as a significant concern, with reported rates reaching as high as 40% to 50% in Pakistan and Turkey, respectively [27,28]. In the present study, a statistically significant (*p* < 0.05) correlation was observed between *E. coli* and its resistance to CIP (r = 0.93). This rate is similar to Daoud et al.’s [25] study, which reported a resistance rate of 25.2% for fluoroquinolones (FQ) in Tunisia. The rise in CIP resistance is a result of overuse of CIP, especially among individuals exposed to FQ in the past six months [25]. Consistent with earlier findings, GEN and AMK (aminoglycosides) have preserved activity against *E. coli* and therefore can be used to treat infections [5,25,26,29]. The increase in resistance of *E. coli* to 3GC has been on the rise, as evidenced by a statistically significant positive correlation (r = 91). Between 2012 and 2019, the frequency of 3GC resistant strains ranged from 12% to 19%. In Tunisia, the prevalence of ESBL-producing *E. coli* has not been determined in a large-scale surveillance study. However, single-center retrospective studies reported an incidence ranging from 3.3% to 20% [26]. *bla*CTX-M-15 gene-mediated ESBLs are the most prevalent ESBL genotype encountered in Tunisian hospitals [30]. Therefore, carbapenems are considered an alternative empiric treatment when there is a high prevalence of 3GC resistance [29,31]. Since 2011, carbapenem resistance (CR) has been identified in clinical isolates of *E. coli*, although it remains uncommon in Tunisia [22]. Colistin remains the last resort for treating severe infections caused by CR organisms [22,29].

*K. pneumoniae* were the second most frequently isolated GNBs which are the leading cause of UTIs, BSIs, and RTIs, as is consistent with another Tunisian study [26]. Over the course of study period, increased resistance has been observed in *K. pneumoniae* isolates against IMP (r = 0.92), FOX (r = 0.72) and CIP (r = 0.63). *K. pneumoniae* isolates have retained susceptibility towards AMK, carbapenems (IMP and ETP) and colistin. The results correlate with the global trend of resistance observed in *K. pneumoniae* [5,29,32,33]. The increase in prevalence of MDR pathogens in healthcare settings resulted in a preference for carbapenems to manage these infections that consequently led to over consumption of carbapenems [34]. A significant increase in CR *K. pneumoniae* (CRKP) was observed over the span of ten years, and this increase was primarily attributed to the presence of OXA-48-like, NDM-type, and KPC-type carbapenemases [34]. To address the challenge posed by CRKP, colistin was regarded as the final treatment choice; however, increased dependence on colistin has led to the development of colistin-resistant *K. pneumoniae* strains [34]. Recently approved combination agents such as ceftazidime–avibactam, imipenem–relebactam, ceftolazone–tazobactam, and meropenem–vaborbactam display activities against CRKP and could serve as better alternatives [29,31]. Unfortunately, only ceftazidime–avibactam and ceftolazone–tazobactam are currently available in Tunisia, and susceptibility testing for these antibiotics is conducted by very few laboratories [35,36].

*P. aeruginosa*, a commonly encountered non-fermentative GNB, is known to cause various infections ranging from UTIs to severe pneumonia and BSIs, especially among patients with compromised immune system and cystic fibrosis [37]. Pus, pulmonary, urine, and blood samples accounted for 30.5%, 28%, 20.6%, and 6% of the total isolates, respectively, which corresponded closely with Abdallah et al.’s study carried out in Tunisia [38]. During the study period, the correlation analysis suggests that resistance of *P. aeruginosa* to IMP has not increased to a statistically significant level. Earlier studies carried out in Tunisia showed the dissemination of VIM-2 carbapenemase [22] and, recently, the emergence of GES-5 and GES-9 carbapenemases [39]. This observation may indicate a rise in MDR rates within the *P. aeruginosa* isolates, posing a significant public health concern that is difficult to treat [31]. Newer combination therapies, such as ceftazidime–avibactam, imipenem–relebactam, and ceftolazone–tazobactam, could serve as an effective strategy to treat these infections, as demonstrated by their high susceptibility in a study conducted by Sastre-Femenia et al. [40].

*A. baumannii*, another essential non-fermentative GNB, contributes to RTIs, UTIs, and BSIs, predominantly sourced from pulmonary, urine, and blood culture samples. Managing infections caused by *A. baumannii* becomes challenging, as this organism shows resistance against multiple classes of antibiotics [31]. The current study reported that the antimicrobial susceptibility rate for all tested antibiotics was below 20%, while the resistance rate to IMP was 85.5% in 2019, which aligns with the findings of another Tunisian study [41]. Similar to the findings in the Nadia et al. study, this report also detected a statistically significant positive correlation (r = 0.95) between *A. baumannii* and its resistance to IMP from 2008 to 2019 [37]. In Tunisia, carbapenem resistance among *A. baumannii* species is generally associated with production of OXA-23, OXA-58, the intrinsic OXA-51 carbapenemases [22], and, recently, VIM-2 carbapenemase [42]. Globally, *A. baumannii* demonstrates a higher level of MDR compared to *P. aeruginosa* and *K. pneumoniae* [29]. Since MDR *A. baumannii* retains susceptibility to colistin, it may serve an option for treating infections caused by this pathogen.

This study offers several notable strengths. Its longitudinal design over two decades provides critical insights into resistance evolution. The large multicenter dataset, comprising over 213,000 isolates from 11 Tunisian hospitals, ensures broad representation. Furthermore, standardized testing based on EUCAST guidelines, with regular internal quality controls, ensures consistency across sites. However, some limitations must be acknowledged. The retrospective design of the study restricts the ability to control for confounding factors and changes in clinical or laboratory practices over time. Patient-level data, such as comorbidities, outcomes, and prior antibiotic use, were not available. In addition, molecular typing of resistance genes was not performed, which would have strengthened our understanding of resistance mechanisms. The gradual expansion of surveillance sites over the study period may also have introduced bias. Finally, although amikacin and colistin remain effective against many MDR isolates, testing for newer antibiotics was limited.

Despite these limitations, this study provides critical baseline data to support national and regional antimicrobial stewardship efforts. Ongoing surveillance, expanded molecular diagnostics, and access to newer antimicrobials are essential to address the rising threat of Gram-negative antimicrobial resistance in Tunisia.

## 4. Conclusions

In conclusion, this study provides a comprehensive 20-year analysis of antimicrobial resistance patterns among major Gram-negative pathogens in Tunisia, using data from the LART surveillance program. The findings reveal a substantial increase in resistance among *E. coli* to third-generation cephalosporins, ciprofloxacin, and ertapenem, and among *K. pneumoniae* to imipenem, cefoxitin, and ciprofloxacin—highlighting the growing threat of extended-spectrum β-lactamase and carbapenemase producers. *A. baumannii* exhibited alarmingly high resistance to most antibiotics, including a dramatic rise in imipenem resistance, while *P. aeruginosa* resistance trends remained relatively stable. These results underscore the urgency of addressing multidrug resistance in clinical settings and provide critical national-level data to inform antibiotic stewardship, empirical treatment protocols, and future AMR containment strategies in Tunisia and similar contexts. From a public health perspective, the study supports the need for strengthened national AMR surveillance; regulatory action to curb antibiotic misuse; and investment in access to newer, effective antimicrobials to mitigate the escalating burden of resistant infections.

## 5. Materials and Methods

### 5.1. Study Sites and Inclusion

This retrospective overview was conducted between January 1999 and December 2019 across several different healthcare facilities in Tunisia that were part of the AMR surveillance network during that time.

During the 20-year study period, a total of 11 Tunisian hospitals participated. During the period 1999–2010, AMR surveillance was carried out in four different university hospitals, three from the Greater Tunis and one from Sfax (regrouping two hospitals). Since 2011, the AMR surveillance was extended to 11 university hospitals. The key healthcare facilities of two major cities in Tunisia, Tunis and Sfax, were part of the surveillance throughout the study period. Greater Tunis and Sfax are two prominent urban areas in Tunisia with distinct socioeconomic and health profiles. As the capital and largest urban agglomeration, Greater Tunis has a significant concentration of the nation’s healthcare resources, accounting for a third of the country’s public hospital beds, with 6679 beds in 2023 [43]. Sfax, Tunisia’s second-largest city, is renowned for its industrial and commercial activities. It serves as a key medical hub for the country and attracts patients from neighboring regions. Sfax hosts prominent university hospitals, like Habib Bourguiba University Hospital and Hedi Chaker University Hospital, with 1814 beds in 2023 [43].

GNB isolates of *E. coli*, *K. pneumoniae*, *P. aeruginosa*, and *A. baumannii* were collected from different samples, including blood cultures, urine, and pulmonary samples, from hospitalized patients and outpatients. Samples were collected using routine methods, and species were identified using conventional methods based on morphological characteristics (colony morphology, Gram staining, and lactose fermentation); and biochemical tests, including oxidase test, API^®^ (BioMérieux, Marcy-l’Etoile, France), or Vitek^®^2 Compact (BioMérieux, Marcy-l’Etoile, France). This process of identifying bacterial species takes approximately 24–48 h.

Isolates from the environment, colonization, and duplicate isolates from the same patient were not included in the analysis. The isolates were obtained from surgery, medicine, gynecology, neonatology, pediatrics, emergency, intensive care, onco-hematology, and outpatient departments.

### 5.2. Antimicrobial Susceptibility Testing

Following the identification of species, antimicrobial susceptibility testing (AST) was performed using disk diffusion method through disc diffusion on Mueller–Hinton agar, according to EUCAST guidelines [44] that were applicable at the time of sample collection. First, a standardized inoculum was prepared by suspending bacterial colonies from a fresh culture in saline to achieve a turbidity equivalent to 0.5 McFarland standard. This suspension was then evenly spread on Mueller–Hinton agar plates using a sterile swab. Antimicrobial disks were placed on the inoculated agar surface within 15 min of inoculation. The plates were incubated at 35 ± 1 °C for 16–20 h aerobically. Antimicrobial agents tested against *E. coli* and *K. pneumoniae* isolates were as follows: amoxicillin, amoxicillin–clavulanic acid, ticarcillin, piperacillin–tazobactam, cefoxitin, cefotaxime, ceftazidime, imipenem, ertapenem, gentamicin, amikacin, colistin, nalidixic acid, ciprofloxacin, and trimethoprim–sulfamethoxazole. Antimicrobial agents tested against *P. aeruginosa* and *A. baumannii* isolates were ticarcillin, ticarcillin + clavulanic acid, piperacillin, piperacillin-tazobactam, ceftazidime, cefepime, imipenem, meropenem, gentamicin, amikacin, ciprofloxacin, fosfomycin, and colistin. For some antibiotics (including tigecycline for *Enterobacterales* other than *E. coli*), the minimum inhibitory concentration (MIC) was determined using the E-test (BioMérieux^®^, Marcy-l’Etoile, France) or MICE^®^ (Oxoid, UK). The broth microdilution method using the UMIC (Biocentric^®^, Bandol, France) was performed for colistin for extensively resistant strains.

After incubation, the diameters of the inhibition zones and the MIC were measured and interpreted according to EUCAST breakpoint tables, using the version at the time of isolate testing over the 21-year study period. Internal quality control (IQC) was carried out weekly by testing the recommended control strains (*E. coli* ATCC25922 and *P. aeruginosa* ATCC 27853).

All the laboratories participating in the surveillance program followed a comparable methodology regarding bacterial species identification, antibiotic susceptibility testing, and interpretation.

### 5.3. Statistical Analysis

Data were entered into Excel spread sheets, and continuous variables were analyzed using descriptive statistics. The evolution of *K. pneumoniae*, *E. coli*, *A. baumannii*, and *P. aeruginosa* resistance over time was represented by line graphs. Spearman rank correlation coefficients were calculated between the years and the resistance variable for each bacterium using R 4.2.1. The results were considered statistically significant if *p*-value was <0.05.

## Figures and Tables

**Table 1:** Overall distribution of isolates categorized by samples from 1999 to 2019 in Tunisia.

Pathogen	Total (*n*)	Urine	Pulmonary	Punctures	Blood Cultures	Pus	Other *
*E. coli*	125,552	107,088	-	1735	4010	7680	5039
*K. pneumoniae*	47,062	27,690	4040	766	6450	6202	1914
*P. aeruginosa*	30,681	6328	8565	1041	1913	9361	3473
*A. baumannii*	10,139	1630	4204	207	1745	1262	1091
Total	213,434	142,736	16,809	3749	14,118	24,505	11,517

* Other: Devices, biopsies, stool, otorhinolaryngology, and neonatal samples. (-) No data available in the surveillance system database.

**Table 2:** Frequency of antibiotic resistance of *E. coli* and *K. pneumoniae* in Tunisia from 1999 to 2019: overall, urine, and blood cultures isolates.

Pathogen	*K. pneumoniae* (*n* = 47,062)						*E. coli* (*n*= 125,552)					
Resistance	Overall (1999–2019)		Urine (2004–2019) *n* = 27,690		Blood Culture (2004–2019) *n* = 6450		Overall (1999–2019)		Urine 1999–2019 *n* = 107,088		Blood Culture (2004–2019) *n* = 4010	
	*n*	(%R)	*n*	(%R)	*n*	(%R)	*n*	(%R)	*n*	(%R)	*n*	(%R)
**AMC**	12,934	(27%)	5917	(25%)	2165	(39%)	23,813	(19%)	20,144	(19%)	547	(21%)
**PTZ**	1911	(4%)	-	-	329	(6%)	1135	(1%)	917	(1%)	49	(2%)
**FOX**	6295	(13%)	3014	(13%)	1092	(20%)	2350	(2%)	1664	(2%)	159	(6%)
**CTX**	18,773	(40%)	8268	(35%)	3353	(60%)	13,598	(11%)	11,269	(11%)	613	(24%)
**CAZ**	18,330	(39%)	8044	(34%)	3230	(58%)	11,602	(9%)	9647	(9%)	522	(20%)
**IMP**	2353	(5%)	750	(3%)	610	(11%)	86	(0%)	35	(0%)	10	(0%)
**ERT**	7530	(16%)	1806	(11%)	936	(25%)	291	(0%)	200	(0%)	22	(1%)
**GEN**	16,808	(36%)	6921	(29%)	2935	(53%)	13,224	(11%)	10,843	(10%)	478	(18%)
**AMK**	4948	(11%)	1929	(8%)	933	(17%)	1766	(1%)	1413	(1%)	78	(3%)
**NAL**	14,564	(31%)	7590	(32%)	2009	(36%)	30,085	(24%)	25,119	(23%)	824	(32%)
**CIP**	14,577	(31%)	7753	(33%)	2049	(37%)	23,572	(19%)	19,915	(19%)	670	(26%)
**SXT**	20,142	(43%)	19,295	41%	18,824	44%	52,731	(42%)	51,476	41%	62,776	50%

*n*, number of resistant isolates; %R, percentage of resistance; amoxicillin–clavulanic acid (AMC); piperacillin–tazobactam (PTZ); cefoxitin (FOX); cefotaxime (CTX); ceftazidime (CAZ); imipenem (IMP); ertapenem (ETP); gentamicin (GEN); amikacin (AMK); nalidixic acid (NAL); ciprofloxacin (CIP); and trimethoprim–sulfamethoxazole (SXT).

**Table 3:** Frequency of antibiotic resistance of *P. aeruginosa* and *A. baumannii* in Tunisia from 1999 to 2019: overall, respiratory, and blood cultures isolates.

Pathogen	*P. aeruginosa* (*n* = 30,681)						*A. baumannii* (*n*= 10,139)					
Resistance	Overall (1999–2019)		Pulmonary Samples (2004–2019) *n* = 8056		Blood Culture (2004–2019) *n* = 1858		Overall (1999–2019)		Pulmonary Samples (2008–2019) *n* = 4204		Blood Culture (2008–2019) *n* = 1745	
	*n*	(%R)	*n*	(%R)	*n*	(%R)	*n*	(%R)	*n*	(%R)	*n*	(%R)
PTZ	2666	(9%)	1039	(14%)	280	(18%)	5413	(53%)	2423	(58%)	848	(49%)
CAZ	4868	(16%)	1319	(18%)	318	(20%)	8245	(81%)	3652	(87%)	1382	(79%)
FEP	2079	(7%)	1010	(14%)	187	(12%)	5443	(54%)	2403	(57%)	896	(51%)
IMP	5798	(19%)	1745	(23%)	394	(25%)	7117	(70%)	3233	(77%)	1176	(67%)
MEM	698	(2%)	269	(4%)	48	(3%)	-	-	-	-	-	-
GEN	7615	(25%)	1554	(21%)	340	(22%)	7576	(75%)	3241	(77%)	1217	(70%)
AMK	4459	(15%)	1013	(14%)	263	(17%)	7049	(70%)	3171	(75%)	1117	(64%)
CIP	6916	(23%)	1750	(23%)	320	(20%)	8320	(82%)	3688	(88%)	1374	(79%)

*n*, number of resistant isolates; %, percentage of resistance; piperacillin–tazobactam (PTZ); ceftazidime (CAZ); cefepime (FEP);; imipenem (IMP); meropenem (MEM); gentamicin (GEN); amikacin (AMK); ciprofloxacin (CIP).

## Data Availability

The data presented in this study are available upon request from the corresponding author.

## Supplementary Materials

The following supporting information can be downloaded at https://www.mdpi.com/article/10.3390/antibiotics14070657/s1, Table S1: Frequency of antibiotic resistance of *E. coli* isolates, including for urines and blood cultures, in Tunisia from 1999 to 2019. Table S2: Frequency of antibiotic resistance of *K. pneumoniae* isolates, including for urines and blood cultures, in Tunisia from 1999 to 2019. Table S3: Frequency of antibiotic resistance of *Pseudomonas aeruginosa* isolates, including for pulmonary samples and blood cultures, in Tunisia from 1999 to 2019. Table S4: Frequency of antibiotic resistance of *Acinetobacter baumannii* isolates, including for pulmonary samples and blood cultures, in Tunisia from 1999 to 2019. Table S5: Frequency of associated antibiotic resistance of third-generation cephalosporin-resistant and ertapenem-resistant *K. pneumoniae* and *E. coli* isolates in Tunisia from 2011 to 2019. Table S6: Frequency of associated antibiotic resistance of imipenem-resistant *P. aeruginosa* and *A. baumannii* isolates in Tunisia from 2011 to 2019.

## Abbreviations

The following abbreviations are used in this manuscript:

AMR	antimicrobial resistance
AMC	amoxicillin–clavulanic acid
AMK	amikacin
AMX	amoxicillin
AST	antimicrobial susceptibility testing
BSI	bloodstream infections
CAZ	ceftazidime
CDC	Centers for Disease Control and Prevention
CIP	ciprofloxacin
CTX	cefotaxime
COL	colistin
EUCAST	European Committee on Antimicrobial Susceptibility Testing
ESBLs	extended-spectrum β-lactamases
ETP	ertapenem
FEP	cefepime
FOS	fosfomycin
FOX	cefoxitin
GEN	gentamicin
GNBs	Gram-negative bacteria
IAI	intra-abdominal infections
IMP	imipenem
IQC	internal quality control
LART	L’AntibioRésistance en Tunisie
MEM	meropenem
MDR	multidrug-resistant
NAL	nalidixic acid
PIP	piperacillin
RTIs	respiratory tract infections
SXT	trimethoprim–sulfamethoxazole
TIC	ticarcillin
TIM	ticarcillin–clavulanic acid
TZP	piperacillin-tazobactam
UTIs	urinary tract infections
3GCs	3rd-generation cephalosporins
