# Supplementary material for: Historical Overview of the Evolution of Multidrug-Resistant Gram-Negative Infections in Tunisia from 1999 to 2019
Source: Antibiotics (Basel). 2025 Jun 29;14(7):657. doi: 10.3390/antibiotics14070657 (PMC12291925; doi:10.3390/antibiotics14070657)
Supplement: Supplementary file 1 [file antibiotics-14-00657-s001.zip › antibiotics-3656142-supplementary.pdf]

Supplementary data

Table S1: Frequency of antibiotic resistance of *E. coli* isolates including for urines and blood cultures in Tunisia from 1999 to 2019

Escherichia coli																					
	**1999**	**2000**	**2001**	**2002**	**2003**	**2004**	**2005**	**2006**	**2007**	**2008**	**2009**	**2010**	**2011**	**2012**	**2013**	**2014**	**2015**	**2016**	**2017**	**2018**	**2019**
**AMC(% R+I)**																					
Overall	25.6	38.9	38.2	38.6	48.3	31.7	35	34.4	26.8	24.4	34.7	28.1	32	39.9	42.2	32	34.4	29.7	27.1	32	35.8
Urine	38.4	30.4	37.3	39	48.7	32.6	36.6	35	29.5	24.8	27.9	28.6	36	38.7	37.9	31.7	33.3	29.8	26.5	23.6	36.1
Blood cultures	NR	NR	NR	NR	NR	NR	NR	NR	NR	33.3	33.3	32.6	42.2	44.9	40.8	45.3	45.6	31.9	41.2	48.1	46.7
**FOX(% R+I)**																					
Overall	1.9	1.9	2.8	1.4	4.5	3.9	3.6	3.9	2.2	4.3	3.2	1.9	2.2	2.9	3.5	3	2.7	3.2	2.9	3.39	4.16
Urine	1.6	1.1	3.1	3	4.2	3.9	3.6	4	2.4	2.8	3.3	1.9	2	2.7	2.4	2.3	2.6	3	2.4	2.78	3.45
Blood cultures	NR	NR	NR	NR	NR	NR	NR	NR	NR	3.7	1.8	1.4	38.3	4.7	5.4	6	7.3	4.4	9.5	6.4	14.3
**CTX(% R+I)**																					
Overall	3.8	2.4	3.6	2.7	5.7	5.4	5.8	7.6	6.5	8	7.9	9.2	6.2	11.8	15.1	17.3	21.9	20.4	19.7	18	18.8
Urine	2.2	3.7	3	3	4.8	4.5	5.4	7	6.3	7.1	7.1	8.5	9.5	10.5	13.8	16.9	21.5	19.9	18.4	17.2	17.8
Blood cultures	NR	NR	NR	NR	NR	NR	NR	NR	NR	15	14.3	14.9	16.7	19.7	29.9	31.9	33.2	27	30.2	34.9	34.4
**CAZ(% R+I)**																					
Overall	3.8	2.4	3.6	2.7	5.7	5.4	5.8	7.6	6.5	8	7.9	9.2	6.2	11.8	15.1	16.9	21.2	20	18.9	17.3	18
Urine	2.2	3.7	3	3	4.8	4.5	5.4	7	6.3	7.1	7.1	8.5	9.5	10.5	13.5	16.5	20.8	19.5	17.8	16.7	15.5
Blood cultures	NR	NR	NR	NR	NR	NR	NR	NR	NR	15	14.3	14.9	16.7	19.7	30.3	30.1	29.8	24.5	29.2	31.7	34.7

Percentage of resistance (%), not recorded in the antimicrobial resistance surveillance system database (NR), Resistant (R), Intermediate susceptibility (I), amoxicillin (AMX), amoxicillin - clavulanic acid (AMC), ticarcillin (TIC), piperacillin – tazobactam (TZP), cefoxitin (FOX), cefotaxime (CTX), ceftazidime (CAZ), imipenem (IMP), ertapenem (ETP), gentamicin (GEN), amikacin (AMK), colistin, nalidixic acid (NAL), ciprofloxacin (CIP), and trimethoprim-sulfamethoxazole (SXT)

Table S1 (suite 1) : Frequency of antibiotic resistance of *E. coli* isolates including for urines and blood cultures in Tunisia from 1999 to 2019

Escherichia coli																					
	**1999**	**2000**	**2001**	**2002**	**2003**	**2004**	**2005**	**2006**	**2007**	**2008**	**2009**	**2010**	**2011**	**2012**	**2013**	**2014**	**2015**	**2016**	**2017**	**2018**	**2019**
**IMP(% R+I)**																					
Overall	0	0	0	0	0	0	0	0	0	0	0	0	0.04	0.1	0.4	0.2	0.2	0.4	0.2	0.2	0.35
Urine	0	0	0	0	0	0	0	0	0	0	0	0	0.01	0.09	0.3	0.1	0.1	0.3	0.1	0.09	0.18
Blood cultures	NR	NR	NR	NR	NR	NR	NR	NR	NR	0	0	0	0	0	0.9	0.5	0.5	0.8	2.1	1.68	1.08
**ETP(% R+I)**																					
Overall	NR	NR	NR	NR	NR	NR	NR	NR	NR	NR	NR	NR	0.6	0.4	0.7	0.5	0.4	0.6	0.5	0.73	0.99
Urine	NR	NR	NR	NR	NR	NR	NR	NR	NR	NR	NR	NR	0.6	0.4	0.5	0.4	0.4	0.5	0.4	0.5	0.73
Blood cultures	NR	NR	NR	NR	NR	NR	NR	NR	NR	NR	NR	NR	0.4	0	1.9	0.5	1	1.3	3.8	3.19	1.91
**GEN(% R+I)**																					
Overall	4.5	4.5	5.2	5.2	8.5	7.8	8.3	6.2	9.5	10.2	10.5	9.7	9.8	13.6	14.7	15.8	18	16.5	14.2	13	13.3
Urine	5.1	4.9	4.4	5	7.4	7	7.9	9.8	9.7	9.3	10.3	8.5	7.6	12.7	13.8	15.6	18	14.9	13.9	12.3	13.3
Blood cultures	NR	NR	NR	NR	NR	NR	NR	NR	NR	18.2	14.8	12.9	12.9	18.1	26.2	25	23.5	16.9	23.4	25.7	11.5
**AMK(% R+I)**																					
Overall	0.5	0.8	2.2	1.6	4	3.1	3.7	4.5	4.9	4.6	4.4	2.3	2.8	2.5	2.7	3.4	1.3	1.6	1.3	1.64	1.81
Urine	1	1.6	2.5	1	3.5	3	3.6	4.4	5	4.4	4.2	2.3	2.6	2.3	2.2	3.1	2.2	1.6	1.3	1.59	1.68
Blood cultures	NR	NR	NR	NR	NR	NR	NR	NR	NR	10.6	9	3.4	4.1	4.3	3.6	6.8	2.9	4.2	2.3	4.52	4.28

Percentage of resistance (%), not recorded in the antimicrobial resistance surveillance system database (NR), Resistant (R), Intermediate susceptibility (I), amoxicillin (AMX), amoxicillin - clavulanic acid (AMC), ticarcillin (TIC), piperacillin – tazobactam (TZP), cefoxitin (FOX), cefotaxime (CTX), ceftazidime (CAZ), imipenem (IMP), ertapenem (ETP), gentamicin (GEN), amikacin (AMK), colistin, nalidixic acid (NAL), ciprofloxacin (CIP), and trimethoprim-sulfamethoxazole (SXT)

Table S1 (suite 2) : Frequency of antibiotic resistance of *E. coli* isolates including for urines and blood cultures in Tunisia from 1999 to 2019

Escherichia coli																					
	**1999**	**2000**	**2001**	**2002**	**2003**	**2004**	**2005**	**2006**	**2007**	**2008**	**2009**	**2010**	**2011**	**2012**	**2013**	**2014**	**2015**	**2016**	**2017**	**2018**	**2019**
**NAL(% R+I)**																					
Overall	11.9	11.9	12.2	13.7	18.5	17.5	17.2	19.4	21.7	19.6	22.1	22.7	21.3	26.9	30.2	33	33.7	32.8	34.2	33.4	32.8
Urine	11.9	11.6	12	13	17.7	17.8	17.2	18.9	19.2	19.9	22.2	23.3	21.1	25.5	28.9	32.9	34.1	32.8	33.6	32.5	32.7
Blood cultures	NR	NR	NR	NR	NR	NR	NR	NR	NR	25.7	23.8	22.4	26.4	29.5	36.2	35.4	41.4	36.9	35.8	45.2	35.1
**CIP(% R+I)**																					
Overall	7.9	7.9	8.8	8.9	14.6	14	14.2	16.9	16.5	15.3	16.5	17.4	16.7	21.4	23.1	26.1	28	26.6	26.1	26.7	24.3
Urine	8.4	8.6	5.8	9	14	14.1	14.2	20.3	14.5	17.2	16.8	18	16.6	20.2	22.1	26	28.6	26.8	25.8	26.2	25.6
Blood cultures	NR	NR	NR	NR	NR	NR	NR	NR	NR	21.9	20.8	17.7	17.6	25.2	27.2	35	28.7	30.4	27.3	38.9	28
**SXT(% R+I)**																					
Overall	34	34	39.3	40.8	43.5	42.2	47.3	45.6	45.3	42.8	48.7	46.2	39.2	40.5	44	43.9	44.6	43.2	42.9	42.2	39.2
Urine	40.2	39.8	42.6	41	45.4	44.4	49.5	47.3	49.8	43.5	49.9	47.4	39.8	39.6	43.5	42.7	44.9	43.5	42.6	41.9	39.1
Blood cultures	NR	NR	NR	NR	NR	NR	NR	NR	NR	44.5	50	49	36.7	47.6	49.5	49.7	50.3	47.4	49.7	49.7	41.6

Percentage of resistance (%), not recorded in the antimicrobial resistance surveillance system database (NR), Resistant (R), Intermediate susceptibility (I), amoxicillin (AMX), amoxicillin - clavulanic acid (AMC), ticarcillin (TIC), piperacillin – tazobactam (TZP), cefoxitin (FOX), cefotaxime (CTX), ceftazidime (CAZ), imipenem (IMP), ertapenem (ETP), gentamicin (GEN), amikacin (AMK), colistin, nalidixic acid (NAL), ciprofloxacin (CIP), and trimethoprim-sulfamethoxazole (SXT)

Table S2: Frequency of antibiotic resistance of *K. pneumoniae* isolates including for urines and blood cultures in Tunisia from 1999 to 2019

Klebsiella pneumoniae																					
	**1999**	**2000**	**2001**	**2002**	**2003**	**2004**	**2005**	**2006**	**2007**	**2008**	**2009**	**2010**	**2011**	**2012**	**2013**	**2014**	**2015**	**2016**	**2017**	**2018**	**2019**
**AMC (% R+I)**																					
Overall	33.3	25.2	35.7	38.9	52.8	34.7	41.6	47.2	45.9	50.5	46.5	44.9	44	51.4	29	25.6	45.5	41.1	41.1	43.6	49.1
Urine	NR	NR	NR	NR	NR	32.6	39.4	48.7	43.7	49.9	47.6	41.1	38.1	44.2	48.6	37.9	40.2	37.7	37	39	43.9
Blood cultures	NR	NR	NR	NR	NR	43.5	49.1	66.1	65.3	64.9	54.7	62.5	54.5	70.8	72.9	71.1	66.2	47.7	55.8	58.5	65.2
**FOX (% R+I)**																					
Overall	3.3	4.3	9.4	9.9	28.9	4.8	6.3	7.9	6.8	8.3	6.7	8.3	11.3	14.4	17.7	22.2	24.2	24.5	23.1	24.1	33.2
Urine	NR	NR	NR	NR	NR	5.3	5	8.5	6.8	9.5	7.1	8.5	9.1	10.8	14.8	13.5	17	18.4	20.7	35.8	30.3
Blood cultures	NR	NR	NR	NR	NR	3.27	7	8	8.7	8.7	6.2	7.7	14.1	18.1	37	44.8	37.5	29.6	29.2	38.4	47.9
**CTX (% R+I)**																					
Overall	27.9	31.2	36.2	42.4	43.6	44.5	41.2	46.3	51.5	50.5	46.5	44.9	41.3	46.4	47	44.4	41	43.4	43.3	42.9	48.2
Urine	NR	NR	NR	NR	NR	38.6	31.4	43.1	43.3	49.9	47.6	41.4	35.3	36.7	37.4	33.3	30.4	33.9	37.5	35.8	41.3
Blood cultures	NR	NR	NR	NR	NR	64.1	58	66.1	73.2	64.9	54.7	62.5	63.1	68.3	69.4	74.8	66.8	67.3	70.8	64.7	73

Percentage of resistance (%), not recorded in the antimicrobial resistance surveillance system database (NR), Resistant (R), Intermediate susceptibility (I), amoxicillin (AMX), amoxicillin - clavulanic acid (AMC), ticarcillin (TIC), piperacillin – tazobactam (TZP), cefoxitin (FOX), cefotaxime (CTX), ceftazidime (CAZ), imipenem (IMP), ertapenem (ETP), gentamicin (GEN), amikacin (AMK), colistin, nalidixic acid (NAL), ciprofloxacin (CIP), and trimethoprim-sulfamethoxazole (SXT).

Table S2 (suite1) : Frequency of antibiotic resistance of *K. pneumoniae* isolates including for urines and blood cultures in Tunisia from 1999 to 2019

Klebsiella pneumoniae																					
	**1999**	**2000**	**2001**	**2002**	**2003**	**2004**	**2005**	**2006**	**2007**	**2008**	**2009**	**2010**	**2011**	**2012**	**2013**	**2014**	**2015**	**2016**	**2017**	**2018**	**2019**
**CAZ (% R+I)**																					
Overall	27.9	31.2	36.2	42.4	43.6	44.5	41.2	46.3	51.5	50.5	46.5	44.9	41.3	46.4	48.6	43.8	41.7	42.6	43	42.9	49.6
Urine	NR	NR	NR	NR	NR	38.6	31.4	43.1	43.3	49.9	47.6	41.4	35.3	36.7	35.9	33.5	31.8	32.6	37.9	30	41.9
Blood cultures	NR	NR	NR	NR	NR	64.1	58	66.1	73.2	64.9	54.7	62.5	63.1	68.3	71.3	72.7	65.3	62	68.5	64.2	75
**IMP (% R+I)**																					
Overall	0	0	0	0	0	0	1	0.1	0.1	0	0	0.6	2.6	5.6	12.5	12.4	11.5	11.7	12.5	12.6	18.7
Urine	NR	NR	NR	NR	NR	0	0.9	0.06	0.05	0	0	0.7	1.6	3.7	6	6.4	7.1	9.6	9	9	15.5
Blood cultures	NR	NR	NR	NR	NR	0	3.3	0.2	0	0	0	1	4.7	8.7	20.3	23.1	23.3	15.9	22.3	20.5	25.6
**ETP (% R+I)**																					
Overall	NR	NR	NR	NR	NR	NR	NR	NR	NR	NR	NR	NR	5.6	9.7	15.2	18.2	15.8	15.5	15.9	15.5	22.4
Urine	NR	NR	NR	NR	NR	NR	NR	NR	NR	NR	NR	NR	3.7	6.9	11.8	11.7	10.4	12.4	13	11.4	17.8
Blood cultures	NR	NR	NR	NR	NR	NR	NR	NR	NR	NR	NR	NR	16.1	13.8	29.3	33.8	24.7	24.4	25	24.8	33.4
**GEN (% R+I)**																					
Overall	29.5	32.3	36.4	42.3	41.9	45.4	43.7	45.6	44.7	46.9	41.3	47.1	38.9	39.6	41.2	36.9	28.3	32.1	34.7	30.6	39.7
Urine	NR	NR	NR	NR	NR	36.6	31.7	41.3	38.4	46.4	41.4	31.7	30.2	31.9	34.7	26.9	25	27	28.4	18.5	29.1
Blood cultures	NR	NR	NR	NR	NR	63.7	57	65.2	68.5	59.7	52	48.4	56.1	55.1	57.7	63.5	50.6	47.5	53.5	49.8	56.4

Percentage of resistance (%), not recorded in the antimicrobial resistance surveillance system database (NR), Resistant (R), Intermediate susceptibility (I), amoxicillin (AMX), amoxicillin - clavulanic acid (AMC), ticarcillin (TIC), piperacillin – tazobactam (TZP), cefoxitin (FOX), cefotaxime (CTX), ceftazidime (CAZ), imipenem (IMP), ertapenem (ETP), gentamicin (GEN), amikacin (AMK), colistin, nalidixic acid (NAL), ciprofloxacin (CIP), and trimethoprim-sulfamethoxazole (SXT).

Table S2 (suite2) : Frequency of antibiotic resistance of *K. pneumoniae* isolates including for urines and blood cultures in Tunisia from 1999 to 2019

Klebsiella pneumoniae																					
	**1999**	**2000**	**2001**	**2002**	**2003**	**2004**	**2005**	**2006**	**2007**	**2008**	**2009**	**2010**	**2011**	**2012**	**2013**	**2014**	**2015**	**2016**	**2017**	**2018**	**2019**
**AMK (% R+I)**																					
Overall	13.2	20.4	29.7	28.7	28.3	28.2	26.5	27.2	28.5	24.3	17.4	16.6	11.2	10.7	11.4	12	10.7	9	6.6	9.1	18.3
Urine	NR	NR	NR	NR	NR	23.1	18.4	26.8	24.1	21.9	16.4	13.7	9.7	7.5	8.9	9.5	7	8.4	5.5	7.3	13.5
Blood cultures	NR	NR	NR	NR	NR	43.2	39.5	37.8	46.2	34.2	21.9	21.7	19.3	18.9	15	20.8	21.7	8.5	11.4	15.3	28.2
**NAL (% R+I)**																					
Overall	10.2	13	21.6	31	31.9	32.9	37.6	41.2	46.4	35.7	41.9	37.6	31.9	39.9	43.7	37.8	36.7	37.4	35.8	35	45.5
Urine	NR	NR	NR	NR	NR	33	24.9	40.8	46.2	37.4	48	38.8	32.3	37.1	39.4	34.6	33.9	37.5	34.3	32.1	42.4
Blood cultures	NR	NR	NR	NR	NR	28.7	44.5	42	53.5	29.3	42.1	41.6	39.2	50.3	54.3	56	41.9	38.9	40.7	43.5	53.6
**CIP (% R+I)**																					
Overall	4	7.9	20.2	22.3	29.5	31.2	33.4	38.6	41.9	32.5	42.4	34.7	31.8	37.8	41.3	36.8	34.6	36.4	35.8	35.6	37.6
Urine	NR	NR	NR	NR	NR	28.7	22.8	38.2	41.2	35.3	44.3	36.2	31	42.8	37.1	32.9	30.7	34	34.3	32.8	34.1
Blood cultures	NR	NR	NR	NR	NR	26.8	34.9	27.7	49.9	27.4	37.4	42.2	36.1	45.9	51.2	52.5	40.9	34.1	40.7	45.8	46.1
**SXT (% R+I)**																					
Overall	37.6	39.9	41.7	46.5	43.5	48.6	44.4	49.8	44.9	50.8	47.5	46.4	39.2	41.6	40.7	37.5	40.6	38.4	39.8	39.7	41.5
Urine	NR	NR	NR	NR	NR	49.2	37.9	56.1	47.9	61.2	52.6	53.3	39	42.6	37.5	37.9	37	37.3	39	37.5	40.8
Blood Cultures	NR	NR	NR	NR	NR	51.5	55.3	47.2	53.5	45.1	48.7	50.5	39	49.5	45	49.5	44.3	49	45.9	51.1	52.3

Percentage of resistance (%), not recorded in the antimicrobial resistance surveillance system database (NR), Resistant (R), Intermediate susceptibility (I), amoxicillin (AMX), amoxicillin - clavulanic acid (AMC), ticarcillin (TIC), piperacillin – tazobactam (TZP), cefoxitin (FOX), cefotaxime (CTX), ceftazidime (CAZ), imipenem (IMP), ertapenem (ETP), gentamicin (GEN), amikacin (AMK), colistin, nalidixic acid (NAL), ciprofloxacin (CIP), and trimethoprim-sulfamethoxazole (SXT).

Table S3: Frequency of antibiotic resistance of *Pseudomonas aeruginosa* isolates including for pulmonary samples and blood cultures, in Tunisia from 1999 to 2019

Pseudomonas aeruginosa																					
	**1999**	**2000**	**2001**	**2002**	**2003**	**2004**	**2005**	**2006**	**2007**	**2008**	**2009**	**2010**	**2011**	**2012**	**2013**	**2014**	**2015**	**2016**	**2017**	**2018**	**2019**
**TIC (%R+I)**																					
Overall	30	30	36	35	31	38	26	27.5	22.5	24.9	24.7	23.3	32.6	28.7	28.6	26.1	24.1	23.6	20.4	22.8	29.9
Pulmonary	NR	NR	NR	NR	NR	38	26	39	31	43.4	27.1	23.4	35.7	28.4	29.8	26	24.8	26.2	19.5	22.6	23.8
Blood cultures	NR	NR	NR	NR	NR	19	29	32.5	26.5	31.6	22.2	24.3	36.6	30.8	32.5	26.7	33	43.8	29.3	34.7	41
**TIM (%R+I)**																					
Overall	28	27	29	27	30	33	26	27	19.5	22.6	25.4	23.3	33.9	28.7	NR	NR	NR	NR	NR	NR	NR
Pulmonary	NR	NR	NR	NR	NR	32	25	36	29.5	39.9	27.5	23.9	40.7	27.4	NR	NR	NR	NR	NR	NR	NR
Blood cultures	NR	NR	NR	NR	NR	19	28	32.5	26.5	31.5	22.2	22.8	-	29.6	NR	NR	NR	NR	NR	NR	NR
**PIP (%R+I)**																					
Overall	NR	NR	NR	NR	NR	NR	NR	NR	NR	18.6	18.3	16.3	NR	NR	19.2	16.1	14.2	22.5	21.4	23.1	31.8
Pulmonary	NR	NR	NR	NR	NR	NR	NR	NR	NR	26.7	17.9	15.9	NR	NR	22.4	18.5	15.7	22.7	21.5	23.5	28.1
Blood cultures	NR	NR	NR	NR	NR	NR	NR	NR	NR	24.1	14.8	18.5	NR	NR	26.2	16.8	28.9	44.5	28.9	33.3	32.8
**TZP (%R+I)**																					
Overall	NR	NR	NR	NR	NR	NR	NR	NR	NR	15.9	14.2	13.6	NR	NR	17.5	13.9	12.9	18	18.5	20.7	26
Pulmonary	NR	NR	NR	NR	NR	NR	NR	NR	NR	24.4	14.1	16.4	NR	NR	20.7	15	12.9	19.4	17.9	20.7	23.4
Blood cultures	NR	NR	NR	NR	NR	NR	NR	NR	NR	24.1	8.6	15.7	35.9	NR	27.9	11.8	20.9	40.9	28.9	31.17	29.6
**CAZ (%R+I)**																					
Overall	14	11	25	21	26	25	24	20	15.5	16.9	17.7	14.6	25	14.8	16	13.2	12.7	14.9	16.1	16.9	22.3
Pulmonary	NR	NR	NR	NR	NR	32	19	33	27	32.3	20.8	19.1	33.4	18.1	16.2	15.2	16.3	15.4	15.6	16.2	18.9
Blood cultures	NR	NR	NR	NR	NR	16	29	22	20	25.2	16	18.5	27.3	21.1	22.5	13	16.5	39.5	25.8	23	27.62

Percentage of resistance (%), not recorded in the antimicrobial resistance surveillance system database (NR), Resistant (R), Intermediate susceptibility (I), Ticarcillin (TIC), TIC+ clavulanic acid (TIM), piperacillin (PIP), piperacillin – tazobactam (TZP), ceftazidime(CAZ), cefepime (FEP), aztreonam (ATM), imipenem (IMP), meropenem (MEM), gentamicin(GEN), amikacin(AMK), ciprofloxacin (CIP), fosfomycin (FOS)

Table S3 (suite 1): Frequency of antibiotic resistance of *Pseudomonas aeruginosa* isolates including for pulmonary samples and blood cultures, in Tunisia from 1999 to 2019

Pseudomonas aeruginosa																					
	**1999**	**2000**	**2001**	**2002**	**2003**	**2004**	**2005**	**2006**	**2007**	**2008**	**2009**	**2010**	**2011**	**2012**	**2013**	**2014**	**2015**	**2016**	**2017**	**2018**	**2019**
**FEP (%R+I)**																					
Overall	NR	NR	NR	NR	NR	NR	NR	NR	NR	NR	NR	NR	NR	NR	19.4	16.1	18.2	14.8	16.2	17.3	22.5
Pulmonary	NR	NR	NR	NR	NR	NR	NR	NR	NR	NR	NR	NR	NR	NR	25.6	24.4	22.4	16.1	14.4	16.5	19.1
Blood cultures	NR	NR	NR	NR	NR	NR	NR	NR	NR	NR	NR	NR	NR	NR	25.3	11.7	22.1	35.6	25.3	30.9	26.55
**IMP (%R+I)**																					
Overall	14	18	20	27	23	24	18	18	17	21.5	22.1	20.9	25.9	22.5	22.4	21.1	21	23.3	19.9	20.8	25.8
Pulmonary	NR	NR	NR	NR	NR	28	28	32.5	30.5	47.8	34.6	26.6	36.3	26.7	20.7	27	25.8	29.2	25.7	26.1	26.7
Blood cultures	NR	NR	NR	NR	NR	21	25	21	26	29	25.9	19.9	30.9	22.7	26.2	23.2	19.6	42.2	34.8	27.3	28.83
**MEM (%R+I)**																					
Overall	NR	NR	NR	NR	NR	NR	NR	NR	NR	NR	NR	NR	NR	NR	NR	NR	NR	NR	NR	20.4	27.2
Pulmonary	NR	NR	NR	NR	NR	NR	NR	NR	NR	NR	NR	NR	NR	NR	NR	NR	NR	NR	NR	20.5	25.9
Blood cultures	NR	NR	NR	NR	NR	NR	NR	NR	NR	NR	NR	NR	NR	NR	NR	NR	NR	NR	NR	33.3	28.3
**GEN (%R+I)**																					
Overall	48	44	54	54	46	40	20	18.5	19.5	25.7	22.2	17.4	20	18.9	15.6	14	15.1	19.5	18.5	20.6	26.8
Pulmonary	NR	NR	NR	NR	NR	40	41	38.5	26.5	35.2	26.1	17.3	30.5	21.2	14.2	17.2	15.9	22.5	21.1	21	21.9
Blood cultures	NR	NR	NR	NR	NR	26	34	31.5	22.5	25.2	22.2	17.1	19.4	20.4	22.5	11.9	16.4	38.5	25.3	28	27.1
**AMK (%R+I)**																					
Overall	12	16	29	26	28	19	16	17.5	15	22.7	15.6	13.7	17.5	13.4	12.8	11.8	12	15.6	15.6	17.3	22.2
Pulmonary	NR	NR	NR	NR	NR	16	27	23.5	20	35.2	14.6	14.9	27.8	12.6	12.8	14.2	11	18.2	18.4	18.8	19.9
Blood cultures	NR	NR	NR	NR	NR	7	24	16.5	13	22.7	4.9	11.4	16.5	15.1	13.7	15.5	15.2	37.2	22.5	30.3	26.55

Percentage of resistance (%), not recorded in the antimicrobial resistance surveillance system database (NR), Resistant (R), Intermediate susceptibility (I), Ticarcillin (TIC), TIC+ clavulanic acid (TIM), piperacillin (PIP), piperacillin – tazobactam (TZP), ceftazidime(CAZ), cefepime (FEP), aztreonam (ATM), imipenem (IMP), meropenem (MEM), gentamicin(GEN), amikacin(AMK), ciprofloxacin (CIP), fosfomycin (FOS)

Table S3 (suite 2): Frequency of antibiotic resistance of *Pseudomonas aeruginosa* isolates including for pulmonary samples and blood cultures, in Tunisia from 1999 to 2019

Pseudomonas aeruginosa																					
	**1999**	**2000**	**2001**	**2002**	**2003**	**2004**	**2005**	**2006**	**2007**	**2008**	**2009**	**2010**	**2011**	**2012**	**2013**	**2014**	**2015**	**2016**	**2017**	**2018**	**2019**
**CIP (%R+I)**																					
Overall	32	28	32	31	34	26	18	19.5	16.5	23.8	22.2	22.1	26.1	24.7	25.6	25.1	18.8	19.5	19.6	31	26
Pulmonary	NR	NR	NR	NR	NR	34	24	29.5	25	40	21.6	23.8	33.1	24.5	26.7	32.5	21.6	22.6	22.2	27	19.1
Blood cultures	NR	NR	NR	NR	NR	17	28	20	17.5	26.5	12.3	20	23.7	26.9	38.8	15.8	14.1	26.7	19.6	17.95	28.71
**FOS (%R+I)**																					
Overall	NR	NR	NR	NR	NR	NR	NR	NR	NR	NR	NR	NR	NR	NR	21.2	27.8	23.7	24.6	13.3	NR	NR
Pulmonary	NR	NR	NR	NR	NR	NR	NR	NR	NR	NR	NR	NR	NR	NR	36.4	28.7	26.6	19.3	13.2	NR	NR
Blood cultures	NR	NR	NR	NR	NR	NR	NR	NR	NR	NR	NR	NR	NR	NR	17	24.6	27.3	35.3	23.8	NR	NR

Percentage of resistance (%), not recorded in the antimicrobial resistance surveillance system database (NR), Resistant (R), Intermediate susceptibility (I), Ticarcillin (TIC), TIC+ clavulanic acid (TIM), piperacillin (PIP), piperacillin – tazobactam (TZP), ceftazidime(CAZ), cefepime (FEP), aztreonam (ATM), imipenem (IMP), meropenem (MEM), gentamicin(GEN), amikacin(AMK), ciprofloxacin (CIP), fosfomycin (FOS)

Table S4: Frequency of antibiotic resistance of *Acinetobacter baumannii* isolates including for pulmonary samples and blood cultures in Tunisia from 1999 to 2019

Acinetobacter baumannii												
	**2008**	**2009**	**2010**	**2011**	**2012**	**2013**	**2014**	**2015**	**2016**	**2017**	**2018**	**2019**
**TIC(%R+I)**												
Overall	78.6	88.6	75.3	84.4	89	89	86.5	86.2	88	86.9	88.4	85
Pulmonary	95.9	95.3	95	94.5	94.6	93.3	90.7	90.3	96.4	93.4	95.5	93.5
Blood cultures	80.9	78.3	65.6	76.5	85.3	88.1	90.7	87.6	82.6	81.8	84.6	87.1
**TIM(%R+I)**												
Overall	76.9	88.2	75.2	80.1	60.2	NR	NR	NR	NR	NR	NR	NR
Pulmonary	90	95.3	95	93	68.7	NR	NR	NR	NR	NR	NR	NR
Blood cultures	78	76	64	71.5	48.3	NR	NR	NR	NR	NR	NR	NR
**PIP(%R+I)**												
Overall	NR	NR	NR	NR	NR	89.7	85.5	86.2	91.2	90.9	93.6	93.8
Pulmonary	NR	NR	NR	NR	NR	91.8	89.5	90.3	96.2	96.8	97.4	95.9
Blood cultures	NR	NR	NR	NR	NR	87	91.6	87.6	87.6	80.5	88.2	91.8
**TZP(%R+I)**												
Overall	NR	NR	NR	NR	NR	86.2	83.9	84.4	87.5	87.9	89.8	90.8
Pulmonary	NR	NR	NR	NR	NR	89.7	89.7	88.3	97.4	95.4	95.8	93.5
Blood cultures	NR	NR	NR	NR	NR	79.7	88.5	80	78.1	77.7	86	88.8
**CAZ(%R+I)**												
Overall	41	90.1	74.6	87.2	91	92.8	86.3	90.6	90.8	90.2	89.5	89.2
Pulmonary	96.6	96.4	93.5	91	91.9	92.5	89.5	89.1	96.8	95.4	95.3	92.4
Blood cultures	83.7	76.2	88	75	89.1	89.4	90.6	89	86.2	80.5	82.1	85.8

Percentage of resistance (%), not recorded in the antimicrobial resistance surveillance system database (NR), Resistant (R), Intermediate susceptibility (I), Ticarcillin (TIC), TIC+ clavulanic acid (TIM), piperacillin (PIP), piperacillin – tazobactam (TZP), ceftazidime(CAZ), cefepime (FEP), aztreonam (ATM), imipenem (IMP), meropenem (MEM), gentamicin(GEN), amikacin(AMK), ciprofloxacin (CIP)

Table S4 (suite) : Frequency of antibiotic resistance of *Acinetobacter baumannii* isolates including for pulmonary samples and blood cultures in Tunisia from 1999 to 2019

Acinetobacter baumannii												
	**2008**	**2009**	**2010**	**2011**	**2012**	**2013**	**2014**	**2015**	**2016**	**2017**	**2018**	**2019**
**FEP(%R+I)**												
Overall	NR	NR	NR	NR	NR	93.9	88.8	90.3	90.4	85.8	86.3	86.8
Pulmonary	NR	NR	NR	NR	NR	92.2	92.2	77.7	96.2	91	94.1	93.1
Blood cultures	NR	NR	NR	NR	NR	82.7	98.1	90.2	85.2	82	80.8	83.5
**IMP(%R+I)**												
Overall	44.9	64.5	51.4	60.7	79.5	81.3	79.9	80.8	83	83.6	84.4	85.5
Pulmonary	64	86.9	80.1	83	84.5	84.9	86.9	94.1	90.9	91.3	92.6	93.1
Blood cultures	39.9	61.8	64.8	63.2	78.8	76.7	78.7	86.1	78.1	79.2	80.7	82.4
**GEN(%R+I)**												
Overall	73.4	78.5	61.6	66.6	72.5	73.4	75.3	76.3	83	80.3	81.3	83
Pulmonary	89.3	83	84.3	80	78.3	68.8	79.8	81.5	92.1	87.8	88.3	87.7
Blood cultures	75.2	77.3	71.8	64.2	70.1	75.2	78.2	80.2	73.5	79.6	69.6	80.3
**AMK(%R+I)**												
Overall	60.2	64.5	61.4	68.4	70.7	64.3	68.9	67.9	75.7	73	81	81.3
Pulmonary	72	67.1	82.2	87	81.6	63.3	75	70.3	84.6	83.5	90.9	87.9
Blood cultures	60	71	55.8	53	71.1	68	75	63.7	67.3	67.3	74.3	79.3
**CIP(%R+I)**												
Overall	60.8	87.7	74.3	80.4	78.7	89.1	86	85.5	86	87.6	76.9	89.5
Pulmonary	93.3	95.8	92.9	93	91.7	89.4	90.7	80.3	95.9	95.1	94.9	95.2
Blood cultures	83.7	62	45.4	75	80.9	85.6	84	82.4	79.6	84.3	81.2	88.3

Percentage of resistance (%), not recorded in the antimicrobial resistance surveillance system database (NR), Resistant (R), Intermediate susceptibility (I), Ticarcillin (TIC), TIC+ clavulanic acid (TIM), piperacillin (PIP), piperacillin – tazobactam (TZP), ceftazidime(CAZ), cefepime (FEP), aztreonam (ATM), imipenem (IMP), meropenem (MEM), gentamicin(GEN), amikacin(AMK), ciprofloxacin (CIP)

Table S5: Frequency of associated antibiotic resistance of third generation cephalosporin resistant and ertapenem resistant *K. pneumoniae and E. coli* isolates in Tunisia from 2011 to 2019

Year		2011		2012		2013		2014		2015		2016		2017		2018		2019	
Isolate species		Ec	Kp	Ec	Kp	Ec	Kp	Ec	Kp	Ec	Kp	Ec	Kp	Ec	Kp	Ec	Kp	Ec	Kp
**Antibiotic (%R+I)**	**Resistance phenotype**																		
**AMC**	3GC resistant	NR	NR	NR	NR	80.6	91.9	72.4	80.2	68.1	88.4	57.5	80.8	56.7	73.3	63.2	78.1	63.3	84.3
**TZP**	3GC resistant	NR	NR	NR	NR	NR	59.4	NR	58.7	18	64.5	21.9	66.2	36.6	69.1	34.3	66.4	34.7	70.5
**FOX**	3GC resistant	NR	28.4	17.7	29.9	16	35	12	44	8.4	46.7	10.4	49.4	10.7	45.8	12.4	50	16	65.2
	ERT resistant	NR	NR	NR	72.9	52.5	81.2	66.7	91.1	60	94.9	76.1	90.2	85.3	92.7	65.8	94.2	64.9	96.4
**IMP**	3GC resistant	NR	5.6	0.9	11.6	1.4	19.4	1.2	26.7	0.5	23.7	1.2	31.7	0.8	25.9	0.83	28.1	1.29	35.6
	ERT resistant	NR	NR	NR	45.9	50	59.9	50	69	31.8	67.4	48.9	75.2	35	72.9	43.9	74.9	26.2	75.4
**ERT**	3GC resistant	NR	17.7	2.7	19.7	2.1	30.4	2.1	39.9	1.6	34.6	2	40.5	2.2	36.1	3.25	34.9	3.84	44.3
**GEN**	3GC resistant	NR	77.1	51.5	78.4	64.2	79.9	67.8	76.1	59.8	72.8	53.6	68.2	47.2	71.3	45.9	65.2	40.5	66.6
	ERT resistant	NR	NR	NR	80.7	34.4	86.7	42.9	83.9	61.1	86.8	48.6	78.5	64	89.9	53.8	81.1	42.9	81.7
**AMK**	3GC resistant	NR	26.2	12.6	21.6	12.3	21.4	15.6	25.1	8.4	23.3	6.1	20.7	5.6	13.7	5.78	22.8	8.11	33.7
	ERT resistant	NR	NR	NR	30.2	10	28.5	32	37.7	5.3	36.2	15.2	31.3	8.8	25.7	7.84	39.8	9.38	61.5
**CIP**	3GC resistant	NR	59.9	63.8	68.1	66.7	72.8	76.4	69.2	71.2	65.1	66.8	67.2	73	67.6	76	71.8	71.7	70.3
	ERT resistant	NR	NR	NR	87.5	57.1	90.3	78.3	95.4	80	95.3	61.9	93.7	77.8	92	56.5	91.1	63.8	93.4
**SXT**	3GC resistant	NR	70.4	69.7	69.6	72	66.4	73.6	66.4	66.6	74.4	61.3	70.4	66.2	71.9	68.3	72.1	67.1	66.1
	ERT resistant	NR	NR	NR	69.8	63.2	54.3	82.6	65.6	46.2	79.8	67.5	64	81.1	70.3	70.5	64.7	73.7	81.2
**TIG**	ERT resistant	NR	NR	NR	NR	0	NR	7.7	NR	12.5	NR	0	NR	0	2.7	0	18.1	3.9	20.9
**COL**	ERT resistant	NR	NR	NR	NR	NR	NR	NR	NR	NR	NR	NR	1.7	0	10.8	0	19.4	11.8	9.9

E. coli (Ec), *K. pneumoniae* (Kp), third generation cephalosporin (C3G), Percentage of resistance (%), not recorded in the antimicrobial resistance surveillance system database (NR), Resistant (R), Intermediate susceptibility (I), amoxicillin - clavulanic acid (AMC), piperacillin – tazobactam (TZP), cefoxitin (FOX), imipenem (IMP), ertapenem (ETP), gentamicin (GEN), amikacin (AMK), colistine (colistin) , ciprofloxacin (CIP), tigecycline (TIG) and trimethoprim-sulfamethoxazole (SXT).

*The susceptibility of COL was tested by determination of the minimal inhibitory concentration, only for extensively drug resistant isolates

Table S6: Frequency of associated antibiotic resistance of imipenem resistant *P. aeruginosa* and *A. baumannii* isolates in Tunisia from 2011 to 2019

Year	2011		2012		2013		2014		2015		2016		2017		2018		2019	
Antibiotic (%R+I)	PA	AB	PA	AB	PA	AB	PA	AB	PA	AB	PA	AB	PA	AB	PA	AB	PA	AB
TZP	NR	NR	NR	NR	50.3	98.8	50	95.8	46.7	100	56.4	99.4	60.5	100	61.8	100	74.9	99.9
CAZ	61.5	NR	51.6	NR	48.6	98.1	47	94.6	43.3	100	49.9	100	53.1	100	56.9	99	67.6	99.2
FEP	NR	NR	NR	NR	47.5	99.7	48.5	97.5	46.8	100	50.9	100	55.9	99.8	54.9	99.5	63.7	99.7
GEN	58.7	78	56.1	81.2	46	81	45.8	86.2	45.2	90.2	54.1	94.4	52.2	95.4	59.6	89.8	68.5	93.5
AMK	50.8	80	38.9	87.2	34.4	73.3	41.2	79.2	39.1	83.2	46.3	89.2	49.1	87.2	57.1	95.9	59.7	94.8
CIP	66.3	97	59.8	95.5	58.6	98.2	62.2	96.1	45.7	99.3	52	98.6	54.6	99.2	58.5	99.5	68.6	99.7
COL	NR	NR	NR	NR	NR	NR	NR	NR	NR	NR	NR	NR	NR	3	3.6	4.5	11.5	3.4

P. aeruginosa (PA), *A. baumannii* (AB), Percentage of resistance (%), not recorded in the antimicrobial resistance surveillance system database (NR), Resistant (R), Intermediate susceptibility (I), piperacillin – tazobactam (TZP), gentamicin (GEN), amikacin (AMK), colistine (colistin) , ciprofloxacin (CIP)

*The susceptibility of COL was tested by determination of the minimal inhibitory concentration, only for extensively drug resistant isolates
